# Urbanicity, hypothalamic-pituitary-adrenal axis functioning, and behavioral and emotional problems in children: a path analysis

**DOI:** 10.1186/s40359-019-0364-2

**Published:** 2020-02-04

**Authors:** B. E. Evans, J. van der Ende, K. Greaves-Lord, A. C. Huizink, R. Beijers, C. de Weerth

**Affiliations:** 1 Behavioural Science Institute, Radboud University, Montessorilaan 3, 6525 HR Nijmegen, the Netherlands; 2 Centre for Research on Child and Adolescent Mental Health, Karlstad University, Room 1D 349A, Universitetsgatan 2, 651 88 Karlstad, Sweden; 3 Child and Adolescent Psychiatry/Psychology, Erasmus University Medical Center, Wytemaweg 8, 3015 CN Rotterdam, the Netherlands; 4 Section of Clinical Developmental Psychology, Amsterdam Public Health Research Institute, Vrije University Amsterdam, Van der Boechorststraat 1, 1081 BT Amsterdam, the Netherlands; 5 School of Health and Education, University of Skövde, Högskolevägen 1, 541 28 Skövde, Sweden; 6 Donders Institute for Brain, Cognition and Behaviour, Radboud University Medical Center, Kapittelweg 29, 6525 EN Nijmegen, the Netherlands

**Keywords:** Urbanicity, Mental health, Stress, HPA axis, Children

## Abstract

**Background:**

Urbanization is steadily increasing worldwide. Previous research indicated a higher incidence of mental health problems in more urban areas, however, very little is known regarding potential mechanisms underlying this association. We examined whether urbanicity was associated with mental health problems in children directly, and indirectly via hypothalamic-pituitary-adrenal (HPA)-axis functioning.

**Methods:**

Utilizing data from two independent samples of children we examined the effects of current urbanicity (*n* = 306, ages seven to 12 years) and early childhood urbanicity (*n* = 141, followed from birth through age 7 years). Children’s mothers reported on their mental health problems and their family’s socioeconomic status. Salivary cortisol samples were collected during a psychosocial stress procedure to assess HPA axis reactivity to stress, and at home to assess basal HPA axis functioning. Neighborhood-level urbanicity and socioeconomic conditions were extracted from Statistics Netherlands. Path models were estimated using a bootstrapping procedure to detect indirect effects.

**Results:**

We found no evidence for a direct effect of urbanicity on mental health problems, nor were there indirect effects of urbanicity through HPA axis functioning. Furthermore, we did not find evidence for an effect of urbanicity on HPA axis functioning or effects of HPA axis functioning on mental health problems.

**Conclusions:**

Possibly, the effects of urbanicity on HPA axis functioning and mental health do not manifest until adolescence. An alternative explanation is a buffering effect of high family socioeconomic status as the majority of children were from families with an average or high socioeconomic status. Further studies remain necessary to conclude that urbanicity does not affect children’s mental health via HPA axis functioning.

## Background

Living in urban compared to rural areas has been associated with an increased risk of mental health problems [1], and this may be particularly so for children [2]. This effect seems to be independent of other known risk factors such as sex, ethnicity, socioeconomic status (SES) and drug use [3]. Evidence exists for both a causal effect of urbanicity on mental health (e.g. [3]) as well as for the selective migration of individuals at risk for mental health problems toward more urban areas, which seems to be in part genetically driven [4]. Thus, the urbanicity-mental health association seems to be a combination of reciprocal influences between individuals and the wider social environment [5].

Several studies showed that children who lived in a city were more likely to be diagnosed with a psychiatric disorder [6], including autism and attention deficit disorder [7, 8], and were more likely to exhibit psychotic symptoms [9] and behavioral and emotional problems [10] compared to children living in rural areas. Importantly, this association remained when controlling for other major risk factors for mental health problems such as SES, neighborhood social cohesion, and parental symptoms of psychiatric disorders [9, 10].

Strikingly, very little is known about potential mechanisms underlying the association between urbanicity and mental health. Research suggests that a greater demand for social stress processing in cities may explain the association [5, 11]. Social stress can be elicited by, for example, a crowded environment [12], encounters with strangers or unclear dominance order [13], which may increase the threat of social evaluation [14, 15] and defeat [16]. Therefore, children growing up in a city may need to process social stress to a greater degree than their more rural counterparts.

If children in cities are faced with social stress more frequently, it is possible that their underlying biology of stress processing may develop differently than children in rural areas. The hypothalamic-pituitary-adrenal (HPA) axis is one of the main stress systems in humans. It follows a diurnal pattern, with the peak in its end-product cortisol occurring approximately 30 min after awakening (cortisol awakening response; CAR) and subsequently declining. The HPA axis responds to stressors, resulting in increased cortisol levels. Dysregulated HPA axis functioning manifests as flatter daily cortisol curves and exaggerated (hyper-responding) or blunted (hypo-responding) reactivity to stress [17]. Such stress system dysregulation has been linked to poor health outcomes [18, 19].

Some preliminary evidence exists for the hypothesis that social stress processing is different in individuals who live in cities. Results from a neuroimaging study indicated that adults who currently lived in or were brought up in an urban area showed differential limbic brain area responses to psychosocial stress compared to those who lived or grew up in towns and rural areas [20]. Similarly, urban upbringing in adults [21] and current urbanicity in youth [22] were associated with dysregulated HPA axis functioning. These studies provide beginning evidence for the idea that living in an urban area may influence the stress system in youth.

Given that dysregulated stress system functioning has been linked to mental health problems in youth [23, 24], we hypothesized that dysregulated stress system functioning would explain the association between urbanicity and mental health. We tested two path analysis models to examine whether urbanicity was associated with mental health problems: via HPA axis reactivity to a psychosocial stressor (Research question 1; RQ1) and via basal HPA axis functioning (RQ2). We tested these research questions in two independent samples of children in order to examine the effects of current urbanicity (JOiN; Youth Research in the Netherlands sample) and early childhood urbanicity (BIBO; Basal Influences on Babies’ Development sample). The research questions, methodology and analysis plan were peer-reviewed and pre-registered at the Open Science Framework (RQ1: [25]; RQ2: [26]).

## Method

### JOiN sample and study procedure

#### Participants

Participants in the JOiN sample were part of a longitudinal general population study in youth aged six to 18 years [27, 28]. Participants were randomly drawn from the municipal registers of 35 municipalities in the province of South Holland, the Netherlands. In this study, we used data from the first (T1, *N* = 1710) and second (T2, *N* = 990) assessment waves. Due to our current focus on children, we included only those who were between seven and 12 years old at T2 (*N* = 513).

#### Procedure

The Erasmus University Medical Center (EUMC) Ethics Committee approved the study. Parents and children gave written informed consent and assent, respectively, at each wave. T1 took place between December 2003 and April 2005. T2 took place between one and 4 years later, between November 2004 and March 2009. T1 and T2 consisted of parent- and child self-report questionnaires. At T2, children additionally collected four saliva samples at home and participated in a psychosocial stress procedure at a EUMC laboratory. The psychosocial stress procedure began in the early (12:00) or late (15:30) afternoon in order to avoid the circadian morning peaks in cortisol [29]. It commenced with an explanation of the procedure by the test leader after which the children completed questionnaires. After a pre-task rest period, participants completed three social stress tasks (mental arithmetic, public speaking, computer math task) which were characterized by uncontrollability and social-evaluative threat and thus designed to elicit a stress response [14]. The procedure ended with a recovery period and a relaxing nature documentary after which participants were debriefed. Saliva samples were collected six times and perceived stress was assessed five times during the procedure. See Additional file 1 for a detailed description of the psychosocial stress procedure.

#### Measures

##### Mental health problems

Children’s mothers completed the Dutch version of the Child Behavior Checklist (CBCL) 6–18 [30] at T2. All 118 questions pertain to mental health problems. The index for behavioral problems consisted of the mean of the Attention deficit hyperactivity disorder, Oppositional defiant disorder and Conduct disorder symptoms subscales. The index for emotional problems consisted of the mean of the Anxiety, Depression and Somatic symptoms subscales. Cronbach’s alphas were .87 and .77 for behavioral and emotional problems, respectively.

##### Urbanicity

Urbanicity was measured at the neighborhood level. Neighborhoods are defined by Statistics Netherlands as a part of a municipality with a homogenous socioeconomic structure and consist of around 1400 inhabitants on average [31]. Using participants’ home addresses, we extracted data on the neighborhood they lived in for the year that they participated in the psychosocial stress procedure from Statistics Netherlands [32]. Urbanicity is calculated by Statistics Netherlands using the surrounding address density (SAD) which is a continuous measure indicating the degree of human activity in a given area [33]. It is then coded on a scale from 0 (very rural; SAD < 500 addresses per km^2^) to 4 (very urban; > = 2500 addresses per km^2^). This measure is commonly used as a measure of urbanicity in the Netherlands (e.g. [34]).

##### HPA axis reactivity

During the stress procedure, salivary cortisol was collected six times by passive drooling. Taking into account the approximate 20-min delay between activity in the hypothalamus and observable changes in salivary cortisol levels [35], the first two salivary cortisol measurements (Reactivity Cortisol 1; RC1; and RC2) corresponded to cortisol levels during the pre-task period. RC3-RC5 corresponded to cortisol levels during each of the three stressful tasks, and RC6 corresponded to cortisol levels during the recovery period. Saliva samples were kept in a freezer at − 20 degrees Celsius [36] and were collectively sent to the laboratory along with the basal cortisol samples (Technical University Dresden, Dresden, Germany) for assay. Cortisol levels greater than 3 SD above the mean were removed. An area under the curve with respect to increase (AUCi [37]) was calculated as an index of HPA axis reactivity as long as at least one pre-task measurement, plus at least one measurement during the stressful tasks, plus the post-task measurement were available. The lower value of RC1 and RC2 was used in the calculation. In the case of missing cortisol samples, the AUC formula was adjusted to account for time differences between samples.

##### Basal HPA axis functioning

Four tubes were sent to participants’ homes by mail, preserved in the freezer after collection and brought to the university. Participants were instructed to collect samples at awakening (Basal Cortisol 1; BC1), 30 min thereafter (BC2), at 12:00 (BC3) and at 20:00 (BC4) on a normal school day. We calculated three indices of basal HPA axis functioning: the area under the curve with respect to ground (AUCg; 37) as long as BC1, BC2 and BC4 were available), the CAR (BC2 minus BC1) and the decline in cortisol levels from morning to evening (BC1 minus BC4).

##### Perceived stress

Self-reported perceived stress was assessed five times during the psychosocial stress procedure: following the pre-task rest, each of the three stressful tasks, and at the end of the procedure. Participants answered seven questions (e.g. *Can you feel your heart beating? Are you nervous?*) on a visual thermometer and the scores were summed at each time point.

##### Covariates

Neighborhood-level SES was indicated by 16 characteristics of the neighborhood reported by Statistics Netherlands (e.g. average value of housing, proportion of persons with work). A principal components analysis (PCA) of these variables was run in the larger JOiN population (all participants for whom address data was available at T2; *n* = 1105) in order to summarize them, resulting in two components that together explained 63% of the variance: employment (46%) and income (17%). Factor scores of these components were used in the analysis.

We further controlled for family SES, age and sex of the child, and any potential correlates of HPA axis functioning. Family SES was based on the parent-reported (at T1) higher education level of either parent, categorized into low (completed primary or lower secondary education), moderate (completed upper secondary education) and high (completed university) SES, based on the Dutch Education Level Division [38]. Sex and birth date of the child were mother-reported. We examined several potential covariates that could influence HPA axis functioning [22]: ethnicity of the child (Dutch/western immigrant background vs non-western immigrant background; mother-reported), season when cortisol was sampled (spring/summer vs autumn/winter), medicine, alcohol and nicotine use (self- and mother-reported), whether girls had reached menarche and used oral contraceptives (self- and mother-reported), and specifically for HPA axis reactivity: body mass index (measured before the stress procedure), whether children had consumed caffeine, dairy products and engaged in physical exercise on the day of the procedure (self- and mother-reported), and the time of day (12:00 vs 15:30) at which the procedure took place.

#### Available data

We included children in the analyses if they lived in the same neighborhood between T1 and T2. Children who moved to a different neighborhood (*n* = 27) were excluded in order to minimize within-subject variance in neighborhood-level variables. Children were further excluded due to unavailability of neighborhood data (*n* = 6), no or insufficient data on behavioral and emotional problems (*n* = 109), missing samples or insufficient saliva to collect HPA axis indices (AUCi: 11 individuals, AUCg: 33, decline: 27, CAR: 24), and missing data on family SES (*n* = 21). Thus, 306 children had complete data for at least one of the research questions (RQ1: *n* = 256, RQ2: *n* = 282).

#### Statistical analysis

We first examined whether it was necessary to estimate a multilevel model with neighborhood as a second level in the analyses. We calculated intraclass correlations (ICC) using empty models for the outcomes of behavioral and emotional problems separately using the package ‘Multilevel’ [39] in R [40]. The ICC1 indicates the percentage of variance in behavioral and emotional problems that can be explained by group (i.e. neighborhood) membership. The ICC2 is an indication of reliability and should be > .70 [41]. Neighborhood explained very little variance in both behavioral (ICC1 = .08) and emotional (ICC1 = .27) problems, and had very low reliability for both behavioral (ICC2 = .10) and emotional (ICC2 = .31) problems. Therefore, we did not include neighborhood as a level in the analyses and estimated single-level models.

We then determined which potential covariates of HPA axis functioning would be included in the analyses by calculating bivariate correlations between the stress indices and potential covariates. Variables that correlated significantly with the indices of HPA axis functioning were controlled in the respective analyses. For RQ1 we then ascertained whether the psychosocial stress procedure had induced perceived and physiological stress by conducting a repeated-measures analysis of variance (ANOVA) on the perceived stress and cortisol measurements during the procedure.

**Fig. 1:**
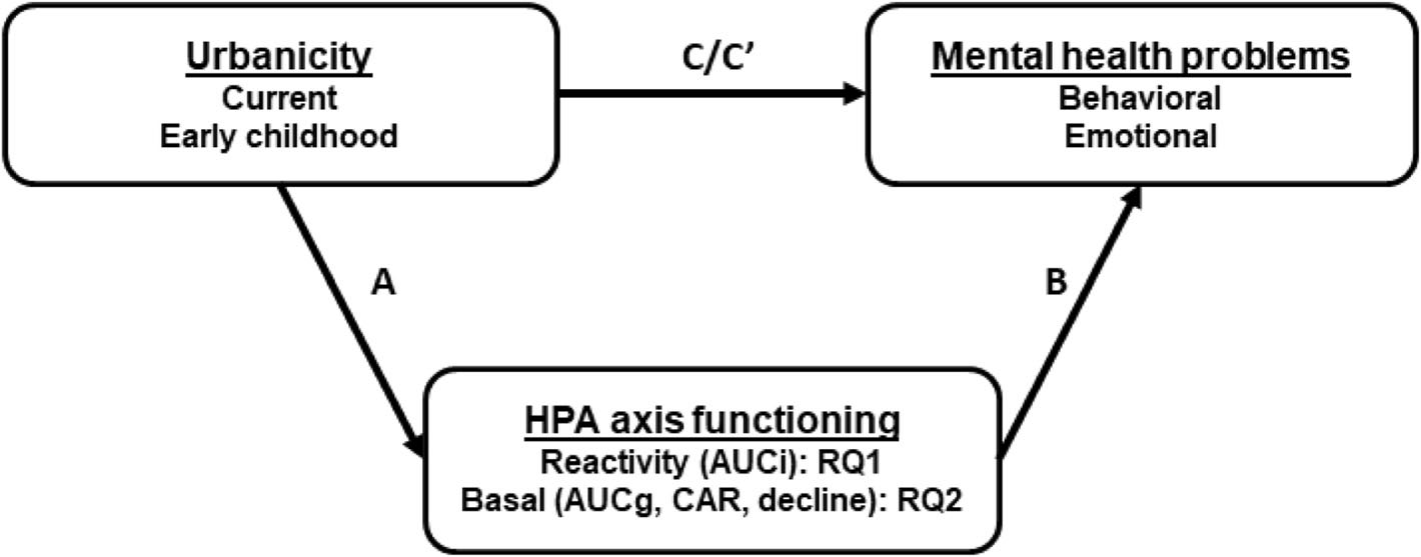
Proposed model of associations between urbanicity, HPA axis functioning and behavioral and emotional problems. *Note*. HPA = hypothalamic-pituiatry-adrenal; AUCi = area under the curve with respect to increase; RQ = research question; AUCg = area under the curve with respect to ground; CAR = cortisol awakening response

We then calculated descriptive statistics of and correlations between all variables, and checked for influential data points using Cook’s distance (criterion *D* < 1). We did this in the sample for whom complete data were available for at least one of the research questions (*N* = 306). The main analyses for both research questions consisted of path models in which the outcome measures of behavioral and emotional problems were estimated separately. We tested whether current urbanicity was associated with behavioral and emotional problems directly (see Fig. 1, path C) and indirectly (path C′) via HPA axis reactivity (AUCi; RQ1) and via basal HPA axis functioning (AUCg, decline, and CAR; RQ2). In addition to the direct and indirect effects of urbanicity, we report the effects of urbanicity on HPA axis functioning (path A) and the effects of HPA axis functioning on behavioral and emotional problems (path B). In all models, we controlled for neighborhood SES, family SES, sex and age of the child, and any covariates related to HPA axis functioning. Effects were considered significant at *p* < .05. Because the direct effects of urbanicity on behavioral and emotional problems were tested twice (in RQ1 and RQ2) in nearly the same samples, these effects were considered significant at *p* < .025. The significance of the indirect effects were estimated using bootstrapping procedures (10,000 samples). We estimated the models with the Process macro [42] in SPSS version 23.

### BIBO sample and study procedure

#### Participants

Children in the BIBO sample were part of an ongoing longitudinal study examining early caregiving and environmental factors and their influences on children’s development and health [43, 44]. Mothers of the children were recruited during pregnancy through flyers distributed to midwife clinics in and around two eastern Dutch cities. The children were all born between January 2006 and July 2007. Follow-up measurements on a sample of 193 children took place multiple times during the first year and approximately once a year thereafter.

#### Procedure

The Faculty Ethics Committee of Radboud University approved the study. Children’s mothers gave written informed consent before starting the study. After the first year, each wave took place around children’s birthdays. Mothers filled in questionnaires when children were 30 months, and four, six and seven years old. When children were six years old, they collected saliva samples at home and participated in a psychosocial stress procedure.

##### Psychosocial stress procedure

Children were tested in a mobile laboratory van parked near their school. The procedure took place between 13:15 and 15:30 and consisted of the Children’s Reactions to Evaluative Stress Test, during which children completed three forced-failure tasks in front of a judge [45]. An experimenter was present during the entire procedure to explain the tasks and give support to the child if they showed signs of distress. After the tasks, children were debriefed and drew and watched movies during a 25-min recovery phase. Salivary cortisol was collected six times. See Additional file 1 for a detailed description of the procedure.

#### Measures

##### Mental health problems

When children were seven years, mothers completed the Dutch version of the CBCL 6–18 [30]. The indices for behavioral and emotional problems were calculated in the same way as in the JOiN study. Cronbach’s alphas were .82 (behavioral problems) and .74 (emotional problems).

##### Urbanicity

Urbanicity was measured in the same way as described in the JOiN sample. We extracted data on the neighborhood the children lived in for each year from birth through age five [32]. The urbanicity score was averaged across these years (calendar years 2006–2011 or 2007–2012) as an index of early childhood urbanicity.

##### HPA axis reactivity

During the stress procedure, saliva was collected six times using eye sponges (BD Visispeare, Waltham, MA [46]) which participants put in their mouths for one minute. During the stress procedure, RC1 and RC2 corresponded to pre-task cortisol levels, RC3 and RC4 to cortisol levels during the stressful tasks, and RC5 and RC6 to cortisol levels during the recovery period. The samples were stored in a freezer and collectively sent to the Endocrinology laboratory of the University Medical Center Utrecht, the Netherlands, for assay. An AUCi was calculated as an index of HPA axis reactivity as long as at least RC1 or RC2, plus RC3 or RC4, plus RC5 or RC6 were available. The lower value of RC1 and RC2 was used in the calculation.

##### Basal HPA axis functioning

Children were instructed to collect eight saliva samples on two consecutive non-school days at awakening (BC1), 11:00 (BC2), 15:00 (BC3), and 19:00 (BC4). The samples were stored at participants’ homes in the freezer until transported to the university, after which they were assayed in the same manner as the cortisol reactivity samples. We calculated two indices of basal HPA axis functioning: AUCg and the decline in cortisol levels (decline; BC1 minus BC4 [47]). Both measures were first calculated per day and then averaged across the two days [48].

##### Covariates

Neighborhood-level SES was indicated by 13 characteristics of the neighborhood reported by Statistics Netherlands. For 2008 and 2011, most income-related variables were missing from the Statistics Netherlands database, therefore we did not use any data from those years. A PCA was carried out for each year (2006–2012, except 2008 and 2011) in all BIBO participants for whom address information was available for the given year. For each year, this resulted in three components: employment, income, and multi-ethnicity, explaining 79–85% of the variance (*M* = 81%, *SD* = 3%). The scores on each of these components were averaged for each participant from birth through age five.

We further controlled for family SES, sex of the child, and potential correlates of HPA axis functioning. Family SES was based on the higher education level of either parent (parent-reported when children were 30 months and four years), categorized into low, moderate and high SES (same categorization as was used in the JOiN study). Potential covariates of HPA axis functioning were: body mass index (measured before the stress procedure), season, and time between BC1 and BC4. All children were of Dutch ethnicity, all children had been asked to refrain from eating, drinking or engaging in physical exercise prior to the psychosocial stress procedure, all sessions took place in the early afternoon, and those that used medicine that might influence cortisol levels were a priori excluded from the analyses, therefore these potential covariates were not tested.

### Available data

Of the 193 children included in the BIBO sample, some were excluded from the analyses due to unavailability of neighborhood data (*n* = 2), no or incomplete data on behavioral and emotional problems (*n* = 22), non-participation in the stress procedure (*n* = 44), or insufficient or unreliable (due to illness or medication use [47, 49]) cortisol data (reactivity: 8, basal: 27). Complete data were available for 141 children (RQ1: 135; RQ2: 115).

### Statistical analysis

The analyses followed the same plan as in the JOiN sample. In the BIBO sample, neighborhood explained very little variance in both behavioral (ICC1 = .25) and emotional (ICC1 = .38) problems, and had very low reliability for both behavioral (ICC2 = .28) and emotional (ICC2 = .42) problems. Therefore, we did not include neighborhood as a level in the analyses and estimated single-level models. Potential covariates of HPA axis functioning were: body mass index, season, and time between sampling of BC1 and BC4. Descriptive statistics were calculated for all participants with data on at least one measure of HPA axis functioning (*N* = 141). In the main analysis, we tested whether early childhood urbanicity was associated with behavioral and emotional problems directly and indirectly via HPA axis reactivity (AUCi; RQ1) and via basal HPA axis functioning (AUCg and decline; RQ2; see Fig. 1). In all models, we controlled for neighborhood SES, family SES, sex of the child, and any covariates related to HPA axis functioning. We did not control for age because all children were tested around their birthday at each wave.

As an additional analysis, we performed a meta-analysis of the results from the models estimated in the JOiN and BIBO samples to examine whether urbanicity (as a general construct) was associated with behavioral and emotional problems directly or indirectly via HPA axis reactivity and basal HPA axis functioning. This analysis was performed in R [50] using the Metafor package [51]. This analysis was included post-hoc in response to a reviewer’s suggestion and was not pre-registered at the Open Science Framework.

## Results

### Preliminary analyses

We confirmed that the psychosocial stress procedure had induced cortisol and perceived stress responses (see Additional file 2). Descriptive statistics of and correlations between all variables are reported in Tables 1 and 2, respectively. The distribution of the urbanicity measure in both samples is depicted in Additional file 3. Descriptive statistics of cortisol samples (basal HPA axis functioning and reactivity) are illustrated in Additional file 4, with the corresponding tables of these statistics in Additional file 5. Histograms of the cortisol measures used in the analysis are depicted in Additional file 6 (JOiN sample) and Additional file 7 (BIBO sample). Bivariate correlations between all potential covariates and the cortisol measures are given in Additional file 8. Means and standard deviations for HPA axis measures differ somewhat between the JOiN and BIBO samples, which is likely due to age differences and/or variations in timing and manner of cortisol sampling. Descriptive statistics at the neighborhood level are given in Additional file 9.

**Table 1:** Descriptive statistics of all variables used in Research questions 1 and 2

	JOiN		BIBO	
	*M* (*SD*) or F(%)	Range	*M* (*SD*) or F(%)	Range
Behavioral problems	0.90 (0.76)	0.00–3.39	0.91 (0.76)	0.00–3.78
Emotional problems	0.59 (0.51)	0.00–2.96	0.42 (0.44)	0.00–2.76
Urbanicity	2.23 (1.36)	0.00–4.00	1.44 (1.08)	0.00–4.00
AUCi (nmol/l)	342.34 (132.60)	66.60–877.40	213.23 (118.59)	37.20–789.00
AUCg (nmol/l)	7497.46 (2116.77)	1359.90–15,945.00	4819.49 (1361.28)	2518.25–10,801.13
Cortisol awakening response	3.18 (6.36)	−22.19-20.90	–	–
Decline	8.84 (5.36)	−10.80-33.79	12.67 (5.03)	−0.35-26.00
SES-Employment	0.10 (0.95)	−3.94-1.78	0.08 (0.92)	−3.05-1.79
SES-Income	−0.12 (0.83)	−2.17-3.01	0.01 (0.89)	−1.69-3.30
SES-Multi-ethnicity	–	–	0.10 (0.90)	−1.79-2.89
Family SES (low/average/high)	10/21/69		0/14/86	
Age at PSP	10.64 (1.40)	7.92–12.92	6.04 (0.13)	5.83–6.75
Sex (male/female)	47/53		51/49	
Season (summer/winter)	44/56		50/50	
Time	*–*	*–*	687.86 (34.01)	570.00–764.00

*Note*. All cortisol measures are indicated in nmol/l. JOiN: *n* = 306 except pertaining to AUCi (*n* = 256), and AUCg, Cortisol awakening response and Decline (*n* = 282); BIBO: *n* = 141 except pertaining to AUCi (*n* = 135), and AUCg and Decline (*n* = 115). *AUCi* Area under the curve with respect to increase, *AUCg* Area under the curve with respect to ground, *SES* Socioeconomic status, *PSP* Psychosocial stress procedure, *Time* Time between basal cortisol 1 and basal cortisol 4

**Table 2:** Correlations between all variables used in Research questions 1 and 2

	1	2	3	4	5	6	7	8	9	10	11	12	13	14	15
1. Urbanicity	–	*.13*	.06	−.07	−.01	−.02	−.04	**−.48**	*−.13*	–	−.05	−.07	.04	.01	–
2. BP	.07	**–**	**.40**	−.02	−.06	−.06	−.02	−.10	**−.19**	–	−.03	−.03	**−.19**	.02	–
3. EP	*.18*	**.28**	–	.04	**.16**	.08	.00	−.05	−.11	–	−.02	.02	*.11*	−.11	–
4. AUCi	.02	−.11	−.06	–	**.44**	.08	.03	.01	−.04	–	−.06	**.19**	*.14*	*−.13*	–
5. AUCg	−.14	−.01	.02	.15	–	**.32**	.01	−.06	*−.12*	–	*−.13*	**.16**	**.19**	**−.23**	–
6. CAR	–	–	–	–	–	**–**	**−.35**	−.02	.09	–	.03	.06	*.14*	−.04	–
7. Decline	*−.19*	−.02	.01	−.01	**.68**	–	–	.11	.10	–	.00	−.11	.08	*.13*	–
8. SES-E	**−.43**	−.16	−.13	.01	.16	–	.13	–	**.40**	–	.05	.08	−.03	−.00	–
9. SES-I	.08	.03	−.00	−.04	.00	–	−.02	*.20*	–	–	**.20**	.01	.00	−.03	–
10. SES-M	**.64**	.02	*.17*	−.01	−.09	–	−.11	−.13	−.09	–	–	–	–	–	–
11. Family SES	.14	.04	−.03	−.11	.10	–	.02	−.01	**.24**	.01	–	.01	.03	−.01	–
12. Age	.00	.09	.03	.08	.04	–	.01	−.08	.03	−.09	.03	–	−.02	−.11	–
13. Sex	−.01	−.07	.04	−.03	.10	–	.08	.13	.09	.06	.15	−.09	–	−.05	–
14. Season	.05	.12	−.07	.02	.02	–	.11	−.09	−.02	−.02	.12	**.37**	.01	–	–
15. Time	.13	−.00	.10	.06	**.25**	–	**.31**	−.05	.03	.11	.01	.04	*−.20*	.00	–

**bold** *= p* < .01; *italics = p* < .05.

*Note*. Correlations are indicated by Spearman’s coefficients. Coefficients in the top right half are from the JOiN study; coefficients in the bottom left half are from the BIBO study. BP = behavioral problems; EP = emotional problems; AUCi = area under the curve with respect to increase; AUCg = area under the curve with respect to ground; CAR = cortisol awakening response; SES = socioeconomic status; E = employment; I = income; M = multi-ethnicity; Time = time between basal cortisol 1 and basal cortisol 4

### Current urbanicity

Results from the path models in the JOiN sample showed that current urbanicity was not associated with behavioral and emotional problems, neither directly (path C) nor indirectly (path C′; see Table 3). Furthermore, current urbanicity was not associated with HPA axis functioning (see Additional file 10) and HPA axis functioning was not associated with behavioral and emotional problems (see Additional file 11).^1^ These models were controlled for neighborhood and family SES, age and sex. Furthermore, season was entered as a control variable as it correlated significantly with HPA axis reactivity and basal HPA axis functioning (see Additional file 8).

**Table 3:** Statistics of direct and indirect effects via HPA reactivity (RQ1) and basal HPA (RQ2) of urbanicity on behavioral and emotional problems in the JOiN and BIBO samples (paths C and C′ in Fig. 1)

	Behavioral problems		Emotional problems	
	Effect (SE)	95% CI	Effect (SE)	95% CI
RQ1: HPA reactivity				
JOiN (n = 256)				
Direct				
Current urbanicity	0.03 (0.04)	−0.04/0.11	0.03 (0.03)	−0.03/0.08
Indirect via				
HPA reactivity				
AUCi	0.00 (0.00)	−0.01/0.01	0.00 (0.00)	−0.00/0.01
BIBO (n = 135)				
Direct				
Early childhood urbanicity	0.04 (0.10)	−0.15/0.23	0.09 (0.05)	−0.02/0.20
Indirect via				
HPA reactivity				
AUCi	−0.00 (0.01)	−0.02/0.02	0.00 (0.01)	−0.01/0.01
Meta-analysis (n = 2)				
Direct				
Urbanicity	0.03 (0.17)	−0.30/0.36	0.05 (0.14)	−0.22/0.32
Indirect via				
HPA reactivity				
AUCi	−0.00 (0.05)	−0.09/0.09	0.00 (0.04)	−0.07/0.08
RQ2: Basal HPA				
JOiN (n = 282)				
Direct				
Current urbanicity	0.05 (0.04)	−0.02/0.13	0.03 (0.02)	−0.01/0.08
Indirect via				
Basal HPA				
AUCg	0.00 (0.00)	−0.01/0.01	−0.00 (0.00)	− 0.01/0.00
CAR	0.00 (0.00)	−0.01/0.01	−0.00 (0.00)	− 0.01/0.00
Decline	−0.00 (0.00)	− 0.01/0.00	0.00 (0.00)	− 0.00/0.00
BIBO (n = 115)				
Direct				
Early childhood urbanicity	0.11 (0.10)	−0.08/0.31	0.12 (0.05)	0.01/0.23
Indirect via				
Basal HPA				
AUCg	0.01 (0.01)	−0.02/0.03	−0.00 (0.01)	− 0.03/0.01
Decline	−0.00 (0.01)	− 0.04/0.02	−0.00 (0.01)	− 0.02/0.01
Meta-analysis (n = 2)				
Indirect via				
Basal HPA				
AUCg	0.00 (0.05)	−0.10/0.10	−0.00 (0.04)	− 0.08/0.08
Decline	−0.00 (0.04)	− 0.09/0.08	−0.00 (0.04)	− 0.07/0.07

*Note*. Statistics of 0.00 are larger than zero, although rounded to two decimal places to avoid long numbers. *HPA* Hypothalamic-pituitary-adrenal, *RQ* Research question, *AUCi* Area under the curve with respect to increase, *AUCg* Area under the curve with respect to ground, *CAR* Cortisol awakening response

### Early childhood urbanicity

Results from the path models in the BIBO sample showed that early childhood urbanicity (from birth through age five years) was not associated with behavioral and emotional problems (at age seven years), neither directly (path C) nor indirectly via HPA axis functioning at age six years (path C′; see Table 3). Furthermore, early childhood urbanicity was not associated with HPA axis functioning (see Additional file 10) and HPA axis functioning was not associated with behavioral and emotional problems (see Additional file 11). These models were controlled for neighborhood and family SES, age and sex. The model in which basal HPA axis activation was investigated as a mediator was controlled for time between BC1 and BC4 (see Additional file 8).

### Urbanicity as a general construct: meta-analysis

A meta-analysis of the results from the models estimated in the JOiN and BIBO samples confirmed that urbanicity was not associated with behavioral and emotional problems directly or indirectly via HPA axis functioning (see Table 3). Urbanicity was not associated with most of the indices of HPA axis functioning, although it was significantly and negatively associated with the AUCg measure of basal HPA axis functioning (see Additional file 10). HPA axis functioning was not associated with behavioral or emotional problems (see Additional file 11).

## Discussion

We examined whether urbanicity was associated with mental health in children directly, and indirectly via HPA axis functioning. We assessed these questions in two independent samples of children to examine the effects of current urbanicity and early childhood urbanicity. We found that urbanicity was not associated with mental health problems directly or indirectly via either HPA axis reactivity or basal HPA axis functioning.

Our finding that urbanicity was not related to mental health was unexpected given previous research. Possibly, this was due to our assessment of sub-clinical symptoms of mental health problems, whereas most previous studies examined the absence versus presence of psychiatric disorders. Newbury and colleagues [9] did find that children who lived in urban areas were more likely to report psychotic symptoms, however, these children were *not* more likely to report symptoms of anxiety, depression or antisocial behavior. Another recent study observed a fairly strong association between urbanicity and teacher-reported mental health problems in Dutch children [10]. However, the sample utilized in that study was from relatively urban neighborhoods, whereas a larger portion of the children in our samples were from more rural neighborhoods. Perhaps associations between urbanicity and mental health are only detectable when mental health problems are sufficiently severe to warrant a diagnoses and/or when children are from more urban neighborhoods.

Our primary aim was to examine potential underlying mechanisms of the presumed urbanicity-mental health link. We hypothesized that, in light of evidence that individuals who live in and grow up in cities must contend with a greater demand for social stress processing [20], the physiological stress system may develop differently in children who live in more urban areas compared to those living in more rural areas. We tested two facets of HPA axis functioning: reactivity and basal functioning, and did not find evidence for either of these as intermediary on the path between urbanicity and mental health. There was no evidence for an association between urbanicity and HPA axis functioning in either of the samples when tested independently, although we did find some evidence for children from more urban areas exhibiting lower basal HPA axis functioning using a meta-analytic approach. One previous study found an association between urban upbringing and HPA axis functioning in adults [21], and another found an association between current urbanicity and HPA axis reactivity in adolescents [22]. Considering that the HPA axis undergoes developmental changes during adolescence [52, 53], it is possible that potential effects of urbanicity on the HPA axis may become more apparent during adolescence. Similarly, drawing on evidence that the duration and severity of maltreatment affects HPA axis functioning [54], it is possible that the children in our sample had not (yet) been exposed to the stress of urban neighborhoods for a long enough time to affect the HPA axis and thereby mental health. This is in line with evidence of a dose-response relationship between urbanicity during childhood and risk for schizophrenia [55].

Alternatively, given that the effects of urbanicity were small, our study may have been under-powered to detect the indirect effects. We used a bootstrapping procedure to increase power, although this may have been insufficient [56]. However, a post-hoc power analysis in G*Power ( [57]; see Additional file 12) showed that we had sufficient power to detect most of the direct effects in the JOiN sample and some of the direct effects in the BIBO sample. In addition, using a meta-analytic approach on the results from both samples confirmed the absence of direct and indirect effects of urbanicity on behavioral and emotional problems.

The lack of significant associations in our study between urbanicity, HPA axis functioning and mental health may also be related to a buffering effect of high quality parental caregiving. In our samples, most children had a high family SES (69 and 86% in the JOiN and BIBO studies, respectively), and very few had a low family SES (10 and 0%, respectively), measured by parental education. Higher parental education has been linked to higher quality parental caregiving [58], and quite some literature suggests that high quality parental caregiving buffers the effects of environmental adversity on child development [59]. Thus, in our samples, higher parental education may have served as a resilience factor, buffering the effects of urbanicity on HPA axis functioning and mental health.

Our results should be considered with regard to some limitations. First, pertaining to the BIBO study, we were unable to include multiple addresses in the models for children who had moved during the study due to the small sample size. Instead, we aggregated neighborhood-level information across their first years of life. However, it is an interesting avenue for future studies to model moving, which would allow a more precise estimation of neighborhood influences on youth. Second, the children in our samples were mostly from families with a higher SES, which limits the generalizability of our findings to children with a lower SES. Third, the JOiN study design was cross-sectional, although we included only children who had lived in the same neighborhood for at least the past year.

## Conclusions

Using data from two independent samples and multiple indices of HPA axis functioning, we did not find evidence for a direct association between urbanicity and mental health, nor did we find evidence for an indirect effect of urbanicity via HPA axis functioning. Considering that developmental changes occur within the biological stress systems during adolescence, and that the impact of urbanicity on HPA-axis functioning may build up over time, it is possible that potential effects of urbanicity on HPA axis functioning do not manifest until a later stage in life. Alternatively, the study may have been under-powered to detect indirect effects, or the high family socioeconomic status of many children may have had a buffering effect. Further studies remain necessary to conclude that urbanicity does not affect children’s mental health via HPA axis functioning.

## Supplementary information

**Additional file 1.** JOiN and BIBO psychosocial stress procedures.

**Additional file 2.** Manipulation check statistics from repeated measures analyses of variance testing whether the psychosocial stress procedures induced physiological and perceived stress.

**Additional file 3.** Histogram depicting the distribution of the urbanicity measure in the JOiN and BIBO samples.

**Additional file 4.** Cortisol levels during the psychosocial stress procedure (A and B) and during the home measurement (C and D) in the JOiN (A and C) and BIBO (B and D) samples.

**Additional file 5.** Descriptive statistics of the raw cortisol values for the JOiN and BIBO samples.

**Additional file 6.** Histograms of cortisol measures as used in the analyses for the JOiN sample.

**Additional file 7.** Histograms of cortisol measures as used in the analyses for the BIBO sample.

**Additional file 8.** Bivariate correlations between cortisol measures used in the analyses and all potential covariates.

**Additional file 9.** JOiN and BIBO sample neighborhood descriptive statistics.

**Additional file 10.** Statistics for the effects of urbanicity on HPA axis functioning in the JOiN and BIBO samples (path A in Figure 1).

**Additional file 11.** Statistics for the effects of urbanicity and HPA axis functioning on behavioral and emotional problems in the JOiN and BIBO samples (paths B and C in Figure 1).

**Additional file 12.** Description of power analysis.

## Data Availability

The data that support the findings of the JOiN study are available from the Erasmus University Medical Center but restrictions apply to the availability of these data, which were used under license for the current study, and so are not publicly available. Data are however available from the authors upon reasonable request and with permission of the Erasmus University Medical Center. The data that support the findings of the BIBO study are available from Radboud University Medical Center but restrictions apply to the availability of these data, which were used under license for the current study, and so are not publicly available. Data are however available from the authors upon reasonable request and with permission of Radboud University Medical Center.

## Abbreviations

ANOVA: Analysis of variance
AUCg: Area under the curve with respect to ground
AUCi: Area under the curve with respect to increase
BC: Basal cortisol
BIBO: Basal Influences on Babies’ Development
CAR: Cortisol awakening response
CBCL: Child Behavior Checklist
EUMC: Erasmus University Medical Center
HPA: Hypothalamic-pituitary-adrenal
ICC: Intraclass correlation
JOiN: Youth Research in the Netherlands
PCA: Principal components analysis
PSP: Psychosocial stress procedure
RC: Reactivity cortisol
RQ: Research question
SAD: Surrounding address density
SES: Socioeconomic status

## Footnotes

1 As an additional test, we ran the models in a subsample of children with a low family SES and the results did not differ from those in the entire sample.
