# Supplementary material for: Urbanicity, hypothalamic-pituitary-adrenal axis functioning, and behavioral and emotional problems in children: a path analysis
Source: BMC Psychol. 2020 Feb 4;8:12. doi: 10.1186/s40359-019-0364-2 (PMC7001285; doi:10.1186/s40359-019-0364-2)
Supplement: Supplementary file 1 — Additional file 1. JOiN and BIBO psychosocial stress procedures. [file 40359_2019_364_MOESM1_ESM.docx]

**Additional file 1**

*JOiN psychosocial stress procedure*

The psychosocial stress procedure took place at a laboratory at the Erasmus University Medical Center in Rotterdam or at temporary laboratories nearer to children’s homes which were set up for the purpose of the study. All sessions took place in the early (12:00) or late (15:30) afternoon in order to avoid the circadian morning peaks in cortisol (1). During the procedure, salivary cortisol was collected six times. An electrocardiogram was also attached in order to monitor participants’ heart rate during the procedure. These data were not used in the current study. Perceived stress was self-reported five times and used to determine whether the procedure was perceived as stressful. The procedure began with an explanation by the test leader of how the procedure would go after which the children completed questionnaires. After a 10-minute pre-task rest period, the social stress tasks began, characterized by uncontrollability and social-evaluative threat and thus designed to elicit a stress response (2). At the beginning of each task, the test leader explained that participants’ performance on the tasks would be compared to others in the same age group. Participants completed three tasks. Each task was performed in front of the test leader who maintained a serious demeanor and gave minimal encouragement during the entire procedure. During the mental arithmetic task (four minutes) they performed serial mental subtraction of seven starting from 100 (for children younger than 12 years) or 23 starting from 1021 for those aged 12. If participants made a mistake the test leader responded: “That is incorrect. Please start again from the beginning”. During the public speaking task (eight minutes mental preparation, six minutes speech) participants had to imagine that they were accused of stealing from the school/workplace cafeteria and gave their response to the accusation. If participants fell silent during the speech part they were urged to continue (e.g. “Please continue”, “If you cannot think of anything else you can tell your story again, but better and with more details”). The public speaking task was recorded on a digital camera. The computer math task (five minutes) consisted of mental number ordering and was performed on a laptop. The session ended with a five-minute recovery period and a relaxing nature documentary (25 minutes) after which participants were debriefed.

*BIBO psychosocial stress procedure*

Children were tested in a mobile laboratory van that was parked near their school (or home in the case of eight children). All children were working in their own classroom prior to the procedure, which took place in the afternoon (between 13:15 and 15:30) in order to avoid the circadian morning peaks in cortisol (1). Teachers were asked not to allow participants to eat, drink, or engage in physical exercise 30 minutes before the procedure began. The procedure consisted of the Children’s Reactions to Evaluative Stress Test (CREST), during which children completed three forced-failure tasks in front of a judge (see 3). An experimenter was also present during the entire procedure to explain the tasks and give support to the child if they showed signs of distress. Before the tasks started, the experimenter showed the children four presents (a tissue, a used eraser, a bubble blower and a kaleidoscope) and asked them to choose which one they liked least and which one they liked most. They were then told that the judge would decide which present they would receive based on their performance on the tasks. During the first task, the child was asked to stand still in front of the judge (for 60 seconds), and not to move otherwise an alarm would go off. Irrespective of the child’s movements, the alarm went off at two pre-programmed times (after 20 and 40 seconds), after which the child was reminded by the judge that it was very important not to move. In the second task, the child listened to a recorded story about animals (three minutes). After each animal name (eight in total), five seconds of silence followed during which the child was asked to imitate the sound of that animal. The judge evaluated the performance by showing either a green or red card. Regardless of the performance, the child was only shown a green card after three of the animal sounds (first, second, and sixth sound). During the third task, the child was asked to make a tower of empty soft drink cans identical to one they were shown by the experimenter. The experimenter uncovered a pyramid of cans (four, three, two, and one can(s) in each layer) which was invisibly glued. The judge told the children it was a very easy task, however, it was actually impossible as the cans kept rolling away. After three minutes, the child was instructed to stop. After the task performance and anticipation of the judge’s evaluation (20 minutes in total), the child was debriefed by showing them how the tasks were rigged. The child then drew and watched movies during a 25-minute recovery phase. Salivary cortisol was collected six times during the procedure.

**References**

1. Kudielka BM, Wüst S. Human models in acute and chronic stress: Assessing determinants of individual hypothalamus–pituitary–adrenal axis activity and reactivity. Stress: The International Journal on the Biology of Stress. 2010;13(1):1-14.

2. Dickerson SS, Kemeny ME. Acute stressors and cortisol responses: A theoretical integration and synthesis of laboratory research. Psychol Bull. 2004 May;130(3):355-91.

3. de Weerth C, Zijlmans MAC, Mack S, Beijers R. Cortisol reactions to a social evaluative paradigm in 5-and 6-year-old children. Stress. 2013 Jan;16(1):65-72.
