# Supplementary material for: Urbanicity, hypothalamic-pituitary-adrenal axis functioning, and behavioral and emotional problems in children: a path analysis
Source: BMC Psychol. 2020 Feb 4;8:12. doi: 10.1186/s40359-019-0364-2 (PMC7001285; doi:10.1186/s40359-019-0364-2)
Supplement: Supplementary file 2 — Additional file 2. Manipulation check statistics from repeated measures analyses of variance testing whether the psychosocial stress procedures induced physiological and perceived stress. [file 40359_2019_364_MOESM2_ESM.docx]

**Additional file 2**

Manipulation check statistics from repeated measures analyses of variance testing whether the psychosocial stress procedures induced physiological and perceived stress.

|  | **Cortisol** | | **Perceived stress** | |
| --- | --- | --- | --- | --- |
|  | *F* | *p* | *F* | *p* |
| **JOiN sample** |  |  |  |  |
| *Main effect* |  |  |  |  |
| Time | 23.25 | < .001 | 50.15 | < .001 |
| *Simple contrasts* |  |  |  |  |
| Pre-task *vs* MAT | 24.11 | < .001 | 39.51 | < .001 |
| Pre-task *vs* PST | 43.45 | < .001 | 72.01 | < .001 |
| Pre-task *vs* CT | 16.35 | < .001 | 4.12 | .04 |
| **BIBO sample** |  |  |  |  |
| *Main effect* |  |  |  |  |
| Time | 18.17 | < .001 |  |  |
| *Simple contrasts* |  |  |  |  |
| Pre-task *vs* Task 1 | 9.15 | .003 |  |  |
| Pre-task *vs* Task 2 | 9.82 | .002 |  |  |

*Note*. All statistics are Greenhouse-Geisser corrected. JOiN sample: main effect of time, cortisol model *df* = 2.68; main effect of time, perceived stress model *df* = 3.17. BIBO sample: main effect of time (cortisol model) *df* = 2.32. For all contrasts: *df* = 1. Pre-task cortisol value pertains to the lower value of RC1 and RC2 in both samples. MAT = mental arithmetic task (RC3); PST = public speaking task (RC4); CT = computer task (RC5); Task 1 = RC3, Task 2 = RC4.
