# Supplementary material for: Urbanicity, hypothalamic-pituitary-adrenal axis functioning, and behavioral and emotional problems in children: a path analysis
Source: BMC Psychol. 2020 Feb 4;8:12. doi: 10.1186/s40359-019-0364-2 (PMC7001285; doi:10.1186/s40359-019-0364-2)
Supplement: Supplementary file 3 — Additional file 3. Histogram depicting the distribution of the urbanicity measure in the JOiN and BIBO samples. [file 40359_2019_364_MOESM3_ESM.docx]

**Additional file 3**

Histogram depicting the distribution of the urbanicity measure in the JOiN and BIBO samples.


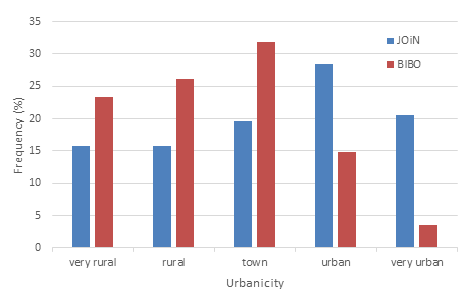


*Note.* For the BIBO sample, the frequencies are rounded to whole numbers for this depiction. In the analyses, the urbanicity measure was used as a continuous score.
