# Supplementary material for: Urbanicity, hypothalamic-pituitary-adrenal axis functioning, and behavioral and emotional problems in children: a path analysis
Source: BMC Psychol. 2020 Feb 4;8:12. doi: 10.1186/s40359-019-0364-2 (PMC7001285; doi:10.1186/s40359-019-0364-2)
Supplement: Supplementary file 4 — Additional file 4. Cortisol levels during the psychosocial stress procedure (A and B) and during the home measurement (C and D) in the JOiN (A and C) and BIBO (B and D) samples. [file 40359_2019_364_MOESM4_ESM.docx]

**Additional file 4**


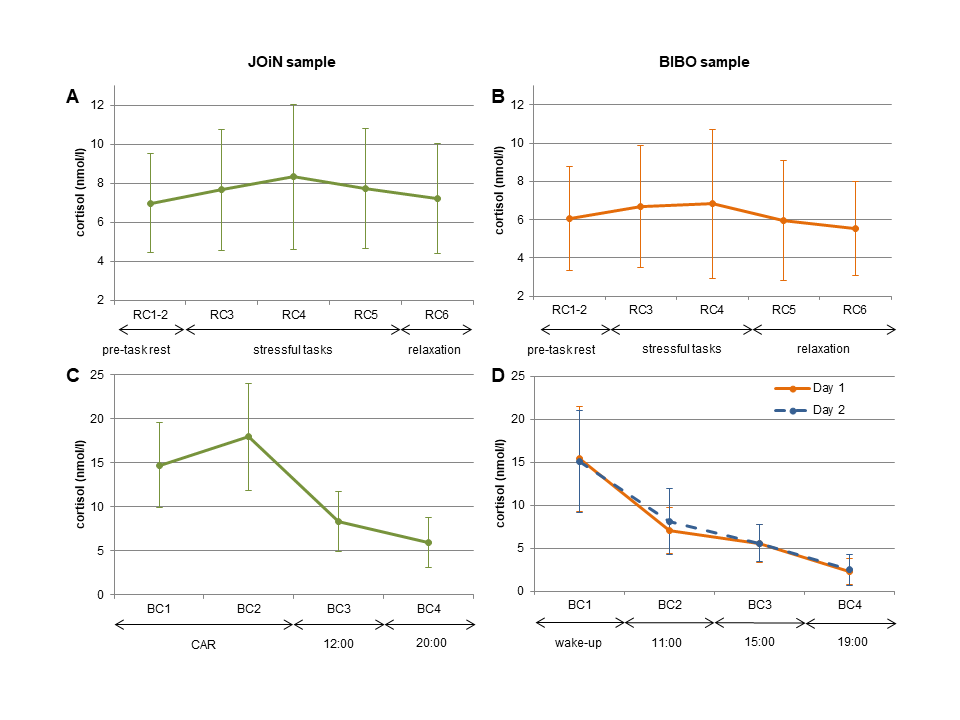


Cortisol levels during the psychosocial stress procedure (A and B) and during the home measurement (C and D) in the JOiN (A and C) and BIBO (B and D) samples.

*Note*. RC = reactivity cortisol; BC = basal cortisol; CAR = cortisol awakening response.
