# Supplementary material for: Urbanicity, hypothalamic-pituitary-adrenal axis functioning, and behavioral and emotional problems in children: a path analysis
Source: BMC Psychol. 2020 Feb 4;8:12. doi: 10.1186/s40359-019-0364-2 (PMC7001285; doi:10.1186/s40359-019-0364-2)
Supplement: Supplementary file 5 — Additional file 5. Descriptive statistics of the raw cortisol values for the JOiN and BIBO samples. [file 40359_2019_364_MOESM5_ESM.docx]

**Additional file 5**

Descriptive statistics of the raw cortisol values for the JOiN and BIBO samples.

|  | **JOiN** *n* = 306 | | | | **BIBO** *n* = 141 | | | |
| --- | --- | --- | --- | --- | --- | --- | --- | --- |
|  | *n* | *M* | *SD* | Range | *n* | *M* | *SD* | Range |
| **RC1** | 258 | 7.65 | 2.67 | 2.10-18.00 | 134 | 6.97 | 3.51 | 1.80-36.00 |
| **RC2** | 257 | 7.35 | 2.71 | 2.10-19.40 | 135 | 6.31 | 2.96 | 1.30-29.00 |
| **RC3** | 257 | 7.64 | 3.10 | 1.50-22.30 | 137 | 6.77 | 3.27 | 1.20-21.00 |
| **RC4** | 256 | 8.33 | 3.70 | 0.80-25.90 | 133 | 6.84 | 3.86 | 1.20-26.00 |
| **RC5** | 257 | 7.79 | 3.24 | 1.30-24.70 | 134 | 5.94 | 3.13 | 1.10-26.00 |
| **RC6** | 259 | 7.31 | 3.06 | 2.00-22.90 | 133 | 5.52 | 2.45 | 1.10-16.40 |
| **BC1a** | 291 | 14.76 | 4.82 | 4.40-34.50 | 126 | 15.40 | 6.02 | 1.70-35.00 |
| **BC1b** |  |  |  |  | 123 | 14.86 | 5.89 | 2.30-34.00 |
| **BC2a** | 295 | 17.82 | 6.07 | 2.00-39.30 | 122 | 6.96 | 2.60 | 2.40-20.00 |
| **BC2b** |  |  |  |  | 116 | 7.94 | 3.70 | 2.00-27.00 |
| **BC3a** | 291 | 8.28 | 3.34 | 0.94-24.50 | 117 | 5.40 | 2.18 | 1.30-14.70 |
| **BC3b** |  |  |  |  | 114 | 5.50 | 2.16 | 1.90-17.40 |
| **BC4a** | 291 | 5.92 | 2.86 | 0.40-25.20 | 122 | 2.27 | 1.46 | 1.00-10.20 |
| **BC4b** |  |  |  |  | 123 | 2.41 | 1.72 | 1.00-15.50 |

*Note.* RC = reactivity cortisol; BC = basal cortisol. In the JOiN sample, RC1 and RC2 indicate pre-task cortisol levels, RC3-RC5 were taken during each of the three stressful tasks, and RC6 was taken after the stressful tasks (recovery). BC1 was taken at awakening, BC2 30 minutes thereafter, BC3 at 12:00 and BC4 at 20:00. In the BIBO sample, RC1 and RC2 indicate pre-task cortisol levels, RC3 and RC4 correspond to cortisol levels during the stressful tasks, and RC5 and RC6 were taken after the stressful tasks (recovery). BC measures were taken on two consecutive days: BC1 at awakening, BC2 at 11:00, BC3 at 15:00 and BC4 at 19:00.
