# Supplementary material for: Urbanicity, hypothalamic-pituitary-adrenal axis functioning, and behavioral and emotional problems in children: a path analysis
Source: BMC Psychol. 2020 Feb 4;8:12. doi: 10.1186/s40359-019-0364-2 (PMC7001285; doi:10.1186/s40359-019-0364-2)
Supplement: Supplementary file 6 — Additional file 6. Histograms of cortisol measures as used in the analyses for the JOiN sample. [file 40359_2019_364_MOESM6_ESM.docx]

**Additional file 6**


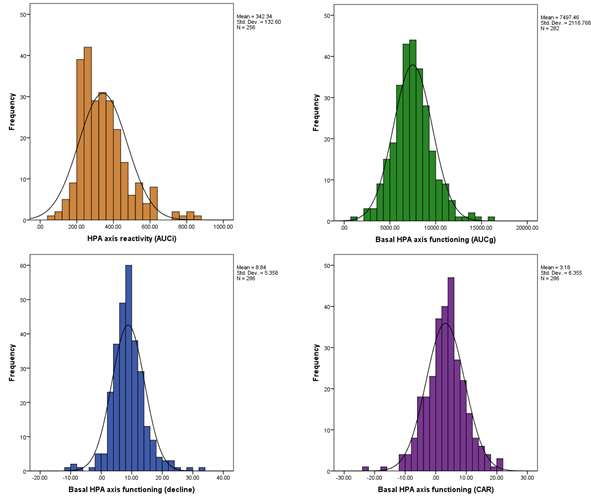


Histograms of cortisol measures as used in the analyses for the JOiN sample.

*Note.* CAR = cortisol awakening response.
