# Supplementary figures and images for: Urbanicity, hypothalamic-pituitary-adrenal axis functioning, and behavioral and emotional problems in children: a path analysis
Source: BMC Psychol. 2020 Feb 4;8:12. doi: 10.1186/s40359-019-0364-2 (PMC7001285; doi:10.1186/s40359-019-0364-2)

**Additional file 7**


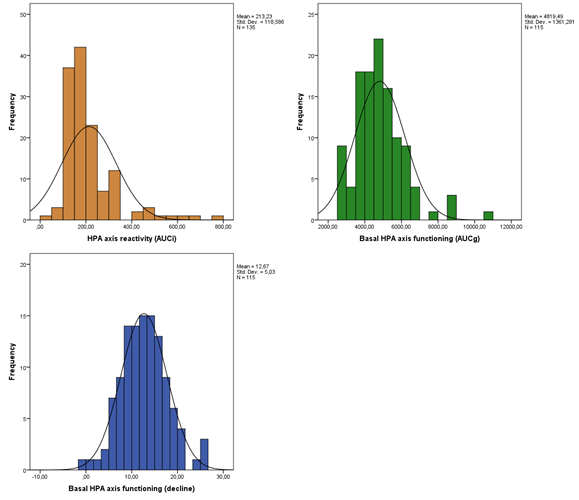


Histograms of cortisol measures as used in the analyses for the BIBO sample.

Supplement: Supplementary file 7 — Additional file 7. Histograms of cortisol measures as used in the analyses for the BIBO sample. [file 40359_2019_364_MOESM7_ESM.docx]
