# Supplementary material for: Urbanicity, hypothalamic-pituitary-adrenal axis functioning, and behavioral and emotional problems in children: a path analysis
Source: BMC Psychol. 2020 Feb 4;8:12. doi: 10.1186/s40359-019-0364-2 (PMC7001285; doi:10.1186/s40359-019-0364-2)
Supplement: Supplementary file 8 — Additional file 8. Bivariate correlations between cortisol measures used in the analyses and all potential covariates. [file 40359_2019_364_MOESM8_ESM.docx]

**Additional file 8**

Bivariate correlations between cortisol measures used in the analyses and all potential covariates.

|  | **1** | **2** | **3** | **4** | **5** | **6** | **7** | **8** | **9** | **10** | **11** | **12** | **13** | **14** | **15** |
| --- | --- | --- | --- | --- | --- | --- | --- | --- | --- | --- | --- | --- | --- | --- | --- |
| 1. AUCi | **-** | **-** | *-* | **-** | **-**.02 | *-.13* | **-**.08 | .11 | .09 | **-**.07 | **-**.06 | **-**.09 | **-**.03 | **-**.11 | **-** |
| 2. AUCg | **-** | **-** | **-** | **-** | .11 | -**.23** | .04 | .04 | .07 | **-** | **-** | **-** | **-** | **-** | **-** |
| 3. CAR | **-** | **-** | **-** | **-** | .08 | **-**.04 | .00 | .10 | .02 | **-** | **-** | **-** | **-** | **-** | **-** |
| 4. Decline | **-** | **-** | **-** | **-** | **-**.03 | *.13* | **-**.08 | **-**.03 | **-**.10 | **-** | **-** | **-** | **-** | **-** | **-** |
| 5. Ethnicity | **-** | **-** | **-** | **-** | **-** | -.03 | .10 | **-**.05 | **-**.12 | .03 | .04 | .02 | .01 | **-**.03 | **-** |
| 6. Season | .02 | .02 | **-** | .11 | **-** | **-** | **-**.03 | .06 | **-**.04 | .09 | .06 | *.15* | **-**.04 | .03 | **-** |
| 7. Medicine use | **-** | **-** | **-** | **-** | **-** | **-** | **-** | .06 | .08 | **-**.03 | *.12* | **-**.08 | .06 | **-**.09 | **-** |
| 8. Alcohol use | **-** | **-** | **-** | **-** | **-** | **-** | **-** | **-** | **.22** | .09 | **-**.05 | .04 | .01 | .00 | **-** |
| 9. Menarche | **-** | **-** | **-** | **-** | **-** | **-** | **-** | **-** | **-** | **.32** | .06 | **-**.09 | **-**.08 | **-**.12 | **-** |
| 10. BMI | .07 | **-** | **-** | **-** | **-** | .11 | **-** | **-** | **-** | **-** | .04 | **-**.07 | **-**.06 | .05 | **-** |
| 11. TD caffeine | **-** | **-** | **-** | **-** | **-** | **-** | **-** | **-** | **-** | **-** | **-** | .03 | .06 | **.20** | **-** |
| 12. TD dairy | **-** | **-** | **-** | **-** | **-** | **-** | **-** | **-** | **-** | **-** | **-** | **-** | **-**.00 | **.22** | **-** |
| 13. TD exercise | **-** | **-** | **-** | **-** | **-** | **-** | **-** | **-** | **-** | **-** | **-** | **-** | **-** | .06 | **-** |
| 14. TD time | **-** | **-** | **-** | **-** | **-** | **-** | **-** | **-** | **-** | **-** | **-** | **-** | **-** | **-** | **-** |
| 15. Time | **-** | **.25** | **-** | **.31** | **-** | .00 | **-** | **-** | **-** | **-**.02 | **-** | **-** | **-** | **-** | **-** |

**bold** *= p* < .01; *italics =* *p* < .05

*Note*. Correlations are indicated by Spearman’s coefficients. Coefficients in the top right half are from the JOiN study; coefficients in the bottom left half are from the BIBO study. AUCi = area under the curve with respect to increase; AUCg = area under the curve with respect to ground; CAR = cortisol awakening response; BMI = body mass index; TD = test day; Time = time between basal cortisol 1 and basal cortisol 4. In the JOiN sample, no participants reported nicotine use or oral contraceptive use.
