# Supplementary material for: Urbanicity, hypothalamic-pituitary-adrenal axis functioning, and behavioral and emotional problems in children: a path analysis
Source: BMC Psychol. 2020 Feb 4;8:12. doi: 10.1186/s40359-019-0364-2 (PMC7001285; doi:10.1186/s40359-019-0364-2)
Supplement: Supplementary file 9 — Additional file 9. JOiN and BIBO sample neighborhood descriptive statistics. [file 40359_2019_364_MOESM9_ESM.docx]

**Additional file 9**

*JOiN sample neighborhood descriptive statistics*

In the JOiN sample, we coded neighborhoods based on geographical boundaries (defined by Statistics Netherlands) as well as time (because data on all neighborhoods were available yearly, and children participated during four different years). Thus, if two participants were from the same geographical neighborhood, but participated in different years, they were considered to be from different neighborhoods (because the neighborhood data vary by year). The final JOiN sample of *n* = 306 children was from 242 neighborhoods in 35 municipalities. Of these 242 neighborhoods, 80% were home to one participant (two participants: 15%, three: 3%, four: 1%, five: 1%). On average, the neighborhoods in the sample had an urbanicity score of 2.4, which is close to the average neighborhood urbanicity score in the Netherlands (3 during the same years as the study). At the individual level, participants were fairly evenly distributed across urban and rural neighborhoods, with 16% living in very rural neighborhoods, 20% living in neighborhoods with an urbanicity score of 2, and 21% living in very urban neighborhoods (see Additional file 3). At the neighborhood level, urbanicity was significantly and negatively correlated with the SES employment component (*r* = -.47, *p* < .001), and negatively correlated with the income component (*r* = -.11, *p* = .09).

*BIBO sample neighborhood descriptive statistics*

In the BIBO sample, we extracted data on the neighborhood for each calendar year between the participant’s birth and age five years. For children who had moved within these years (32% moved once, 6% twice, 1% three times), we used the neighborhood in which they had lived the longest for the following description. The final sample of *n* = 141 children was from 112 neighborhoods in 51 municipalities. Of the 112 neighborhoods, 83% was home to one participant, 11% to two, 4% to three, 1% to four, and 1% to six participants. These neighborhoods were, on average, more rural than in the Netherlands on average (*M* = 1.4 *vs* *M* = 3 in the Netherlands on average). At the individual level, more participants lived in very rural (19%), and average urban/rural neighborhoods (urbanicity score of 2; 21%), and very few lived in very urban neighborhoods (3%; see Additional file 3). At the neighborhood level, urbanicity was significantly and negatively correlated with the SES employment component (*r* = -.46, *p* < .001), not significantly correlated with the income component (*r* = .12, *p* = .22), and significantly and positively correlated with the multi-ethnicity component (*r* = .63, *p* < .001).
