# Supplementary material for: Urbanicity, hypothalamic-pituitary-adrenal axis functioning, and behavioral and emotional problems in children: a path analysis
Source: BMC Psychol. 2020 Feb 4;8:12. doi: 10.1186/s40359-019-0364-2 (PMC7001285; doi:10.1186/s40359-019-0364-2)
Supplement: Supplementary file 10 — Additional file 10. Statistics for the effects of urbanicity on HPA axis functioning in the JOiN and BIBO samples (path A in Figure 1). [file 40359_2019_364_MOESM10_ESM.docx]

**Additional file 10**

Statistics for the effects of urbanicity on HPA axis functioning in the JOiN and BIBO samples (path A in Figure 1).

|  | **HPA reactivity (RQ1)** | | | **Basal HPA (RQ2)** | | | | | | | | |
| --- | --- | --- | --- | --- | --- | --- | --- | --- | --- | --- | --- | --- |
|  | **AUCi** | | | **AUCg** | | | **CAR** | | | **Decline** | | |
|  | Coeff(SE) | *t* | *p* | Coeff(SE) | *t* | *p* | Coeff(SE) | *t* | *p* | Coeff(SE) | *t* | *p* |
| **JOiN** |  |  |  |  |  |  |  |  |  |  |  |  |
| Current urbanicity | -5.21(6.84) | -0.76 | .45 | -19.03(98.64) | -0.19 | .85 | -0.22(0.31) | -0.71 | .48 | 0.12(0.26) | 0.45 | .65 |
| SES-E | 6.77(10.57) | 0.64 | .52 | 31.18(156.09) | 0.20 | .84 | -0.11(0.49) | -0.23 | .82 | **0.84(0.41)** | **2.06** | **.04** |
| SES-I | -5.17(10.88) | -0.48 | .64 | **-348.55(158.68)** | **-2.20** | **.03** | -0.92(0.50) | -1.82 | .07 | 0.17(0.42) | 0.42 | .67 |
| Family SES | -10.02(12.36) | -0.81 | .42 | -292.86(182.14) | -1.61 | .11 | 0.38(0.58) | 0.66 | .51 | -0.05(0.48) | -0.10 | .92 |
| Sex | **37.88(16.32)** | **2.32** | **.02** | **647.53(239.21)** | **2.71** | **.01** | **1.57(0.76)** | **2.08** | **.04** | 1.09(0.63) | 1.73 | .09 |
| Age | **14.31(5.83)** | **2.45** | **.01** | **200.81(86.86)** | **2.31** | **.02** | 0.25(0.27) | 0.91 | .36 | **-0.64(0.23)** | **-2.80** | **.01** |
| Season | -25.66(16.55) | -1.55 | .12 | **-926.63(243.91)** | **-3.80** | **.00** | -0.58(0.77) | -0.75 | .45 | 1.11(0.64) | 1.73 | .08 |
| **BIBO** |  |  |  |  |  |  |  |  |  |  |  |  |
| Early childhood urbanicity | 3.53(14.94) | 0.24 | .81 | -118.41(164.46) | -0.72 | .47 | - | - | - | -0.38(0.61) | -0.63 | .53 |
| SES-E | -5.25(14.44) | -0.36 | .72 | 131.82(167.17) | 0.79 | .43 | - | - | - | 0.53(0.62) | 0.85 | .40 |
| SES-I | -1.28(12.77) | -0.10 | .92 | -141.35(151.86) | -0.93 | .35 | - | - | - | -0.51(0.56) | -0.90 | .37 |
| SES-M | 1.84(15.13) | 0.12 | .90 | -222.36(173.46) | -1.28 | .20 | - | - | - | -0.86(0.64) | -1.33 | .19 |
| Family SES | -30.85(31.32) | -0.98 | .33 | 560.17(410.00) | 1.37 | .17 | - | - | - | 0.56(1.52) | 0.37 | .71 |
| Sex | 9.72(21.25) | 0.46 | .65 | 231.42(256.03) | 0.90 | .37 | - | - | - | 1.63(0.95) | 1.71 | .09 |
| Time | - | - | - | **12.66(3.76)** | **3.37** | **.00** | - | - | - | **0.04(0.01)** | **3.22** | **.00** |
|  | Coeff(SE) | *z* | *p* | Coeff(SE) | *z* | *p* |  |  |  | Coeff(SE) | *z* | *p* |
| **Meta-analysis** (*n* = 2) |  |  |  |  |  |  |  |  |  |  |  |  |
| Urbanicity | -2.47(2.17) | -1.14 | .26 | **-56.29(7.85)** | **-7.17** | **< .001** |  |  |  | -0.03(0.43) | -0.07 | .95 |

*Note*. Sample sizes were *n* = 256 (RQ1) and *n* = 282 (RQ2) in the JOiN sample and *n* = 135 (RQ1) and *n* = 115 (RQ2) in the BIBO sample. Statistics were estimated twice, in the models predicting behavioral problems and emotional problems, respectively, and were very similar. The statistics reported here were taken from the models predicting behavioral problems. HPA = hypothalamic-pituitary-adrenal; RQ = research question; AUCi = area under the curve with respect to increase; AUCg = area under the curve with respect to ground; CAR = cortisol awakening response; SES = socioeconomic status; E = employment; I = income; M = multi-ethnicity; Time = time between the first and fourth basal cortisol measurements (BC1 and BC4).
