# Supplementary material for: Urbanicity, hypothalamic-pituitary-adrenal axis functioning, and behavioral and emotional problems in children: a path analysis
Source: BMC Psychol. 2020 Feb 4;8:12. doi: 10.1186/s40359-019-0364-2 (PMC7001285; doi:10.1186/s40359-019-0364-2)
Supplement: Supplementary file 11 — Additional file 11. Statistics for the effects of urbanicity and HPA axis functioning on behavioral and emotional problems in the JOiN and BIBO samples (paths B and C in Figure 1). [file 40359_2019_364_MOESM11_ESM.docx]

**Additional file 11**

Statistics for the effects of urbanicity and HPA axis functioning on behavioral and emotional problems in the JOiN and BIBO samples (paths B and C in Figure 1).

|  | **Behavioral problems** | | | **Emotional problems** | | |
| --- | --- | --- | --- | --- | --- | --- |
|  | Coeff(SE) | *t* | *p* | Coeff(SE) | *t* | *p* |
| **RQ1: HPA reactivity** |  |  |  |  |  |  |
| **JOiN** (*n* = 256) |  |  |  |  |  |  |
| AUCi | -0.00(0.00) | -0.19 | .85 | -.00(0.00) | -0.43 | .67 |
| Urbanicity | 0.03(0.04) | 0.87 | .39 | 0.03(0.03) | 0.92 | .36 |
| SES-E | -0.02(0.06) | -0.37 | .71 | 0.03(0.04) | 0.68 | .50 |
| SES-I | -0.06(0.06) | -1.01 | .31 | -0.04(0.04) | -0.96 | .34 |
| Family SES | 0.09(0.07) | 1.20 | .23 | -0.00(0.05) | -0.06 | .95 |
| Sex | **-0.33(0.09)** | **-3.48** | **.001** | 0.07(0.07) | 1.04 | .30 |
| Age | 0.00(0.03) | 0.12 | .90 | 0.00(0.02) | 0.12 | .91 |
| Season | 0.03(0.10) | 0.34 | .74 | -0.08(0.07) | -1.18 | .24 |
| **BIBO** (*n* = 135) |  |  |  |  |  |  |
| AUCi | -0.00(0.00) | -0.84 | .40 | 0.00(0.00) | 0.83 | .41 |
| Urbanicity | 0.04(0.10) | 0.41 | .68 | 0.09(0.05) | 1.68 | .10 |
| SES-E | -0.05(0.09) | -0.56 | .58 | -0.03(0.05) | -0.52 | .60 |
| SES-I | 0.05(0.08) | 0.61 | .54 | -0.02(0.05) | -0.38 | .71 |
| SES-M | 0.04(0.10) | 0.42 | .68 | -0.02(0.06) | -0.36 | .72 |
| Family SES | 0.04(0.20) | 0.20 | .84 | -0.05(0.11) | -0.43 | .66 |
| Sex | -0.14(0.14) | -1.02 | .31 | 0.01(0.08) | 0.18 | .86 |
|  | Coeff(SE) | *z* | *p* | Coeff(SE) | *z* | *p* |
| **Meta-analysis** (*n* = 2) |  |  |  |  |  |  |
| AUCi | -0.00(0.02) | -0.02 | .99 | 0.00(0.01) | 0.01 | .99 |
| **RQ2: Basal HPA** |  |  |  |  |  |  |
| **JOiN** (*n* = 282) |  |  |  |  |  |  |
| AUCg | 0.00(0.00) | -1.21 | .23 | 0.00(0.00) | 1.08 | .28 |
| CAR | -0.00(0.01) | -0.10 | .92 | 0.00(0.01) | 0.72 | .47 |
| Decline | -0.00(0.01) | -0.35 | .73 | 0.00(0.01) | 0.10 | .92 |
| Urbanicity | 0.05(0.04) | 1.48 | .14 | 0.03(0.02) | 1.40 | .16 |
| SES-E | 0.02(0.06) | 0.32 | .75 | 0.04(0.04) | 0.92 | .36 |
| SES-I | -0.08(0.06) | -1.38 | .17 | -0.04(0.04) | -0.88 | .38 |
| Family SES | -0.01(0.07) | -0.15 | .88 | 0.02(0.05) | 0.41 | .68 |
| Sex | **-0.26(0.09)** | **-2.81** | **.01** | 0.08(0.06) | 1.26 | .21 |
| Age | -0.00(0.03) | -0.05 | .96 | -0.00(0.02) | -0.21 | .83 |
| Season | 0.03(0.09) | 0.32 | .75 | -0.09(0.06) | -1.47 | .14 |
| **BIBO** (*n* = 115) |  |  |  |  |  |  |
| AUCg | -0.00(0.00) | -0.73 | .47 | 0.00(0.00) | 0.10 | .92 |
| Decline | 0.01(0.02) | 0.47 | .64 | 0.01(0.01) | 0.68 | .50 |
| Urbanicity | 0.11(0.10) | 1.17 | .25 | 0.12(0.05) | 2.14 | .03 |
| SES-E | -0.02(0.10) | -0.21 | .84 | 0.02(0.06) | 0.28 | .78 |
| SES-I | 0.08(0.10) | 0.93 | .36 | 0.04(0.05) | 0.87 | .39 |
| SES-M | 0.01(0.10) | 0.09 | .93 | 0.01(0.06) | 0.15 | .88 |
| Family SES | 0.27(0.25) | 1.09 | .28 | -0.03(0.14) | -0.22 | .82 |
| Sex | -0.16(0.15) | -1.03 | .30 | -0.04(0.09) | -0.42 | .68 |
| Time | 0.00(0.00) | 0.91 | .37 | 0.00(0.00) | 0.78 | .44 |
|  | Coeff(SE) | *z* | *p* | Coeff(SE) | *z* | *p* |
| **Meta-analysis** (*n* = 2) |  |  |  |  |  |  |
| Decline | 0.00(0.08) | 0.01 | .99 | 0.00(0.06) | 0.05 | .96 |

*Note*. Statistics of 0.00 are larger than zero, although rounded to two decimal places to avoid long numbers. HPA = hypothalamic-pituitary-adrenal; RQ = research question; AUCi = area under the curve with respect to increase; SES = socioeconomic status; E = employment; I = income; M = multi-ethnicity; AUCg = area under the curve with respect to ground; CAR = cortisol awakening response; Time = time between the first and fourth basal cortisol measurements (BC1 and BC4). The statistics for the effects of AUCg on behavioral and emotional problems were very small (< 0.000), therefore the meta-analytic approach could not be used for this measure.
