# Supplementary material for: Urbanicity, hypothalamic-pituitary-adrenal axis functioning, and behavioral and emotional problems in children: a path analysis
Source: BMC Psychol. 2020 Feb 4;8:12. doi: 10.1186/s40359-019-0364-2 (PMC7001285; doi:10.1186/s40359-019-0364-2)
Supplement: Supplementary file 12 — Additional file 12. Description of power analysis. [file 40359_2019_364_MOESM12_ESM.docx]

**Additional file 12**

*Description of power analysis*

A post-hoc power analysis was executed using G*Power (4) in the JOiN and BIBO samples separately. Power for multiple linear regression was estimated based on the sample size, an alpha error of .05, the total number of predictors (6 in the BIBO sample, 7 in the JOiN sample) and the R^2^ of the estimated models. The effect size (f^2^) was calculated as R^2^/(1-R^2^). In the BIBO sample, power estimates ranged from .26 to .63 (*M* = .42. *SD* = .17), and in the JOiN sample, from .31 to .99 (*M* = .70, *SD* = .25).

4. Faul F, Erdfelder E, Buchner A, Lang A-G. Statistical power analyses using G* Power 3.1: Tests for correlation and regression analyses. Behavior research methods. 2009;41(4):1149-60.
